# Percutaneous Endoscopic Interlaminar Discectomy via a Modified Inferolateral Margin of the L5 Pedicle Projection for L5/S1 Central Disc Herniation: A Retrospective Study

**DOI:** 10.3390/jcm15145355

**Published:** 2026-07-08

**Authors:** Chenxing Huang, Bin Xie, Zhuangzhuang Tan, Jiajun Cheng, Xiaoteng Feng, Zhenghao Huang, Zhaojun Cheng, Gengyang Shen, Hui Ren, Jingjing Tang, Xiaobing Jiang

**Affiliations:** Department of Spine Surgery, The Second Affiliated Hospital of Guangzhou Medical University, Guangzhou 510260, China

**Keywords:** percutaneous endoscopic interlaminar discectomy, central disc herniation, modified trajectory

## Abstract

**Objective**: To evaluate the feasibility and short-term outcomes of a modified percutaneous endoscopic interlaminar discectomy (PEID) trajectory via the inferolateral margin of the L5 pedicle projection for central disc herniation at L5/S1. **Methods**: From June 2023 to July 2025, a consecutive cohort of 70 patients diagnosed with L5/S1 lumbar disc herniation (LDH) undergoing modified projection PEID was enrolled. Based on preoperative MRI (magnetic resonance imaging) evaluation of herniation types, they were divided into a central herniation group (*n* = 32) and a non-central herniation group (*n* = 38). Clinical outcomes and radiographic parameters were subsequently compared between the groups. **Results**: Seventy patients were included (38 non-central and 32 central). Baseline characteristics were comparable between the two groups except for a higher prevalence of bilateral symptoms in the central herniation group (*p* = 0.036). The central herniation group exhibited significantly shorter operative time (*p* = 0.027), reduced intraoperative blood loss (*p* = 0.047), and lower haemoglobin drop (*p* = 0.033). Multivariable linear regression confirmed that central herniation was independently associated with improved surgical efficiency. Both groups demonstrated significant postoperative improvements in VAS (Visual Analogue Scale) and JOA (Japanese Orthopaedic Association) scores. Although the non-central herniation group showed a higher JOA score on postoperative day 1 (*p* = 0.047), no significant differences were observed at 3 months or the final follow-up. The modified MacNab satisfaction rates were comparable (*p* = 0.411), with a similarly low complication rate in both groups. **Conclusions**: The modified PEID trajectory via the inferolateral margin of the L5 pedicle projection is feasible for central L5/S1 disc herniation and is associated with reduced operative time and decreased intraoperative blood loss.

## 1. Introduction

Lumbar intervertebral disc protrusion has gradually become an important factor affecting global health. When conservative treatment has been ineffective for more than three months, surgical intervention often serves as an effective solution to relieve patients’ pain [1,2,3]. Endoscopic spine surgery has become the mainstream surgical approach for treating lumbar disc herniation because of its advantages of minimal trauma, rapid recovery, and short hospital stays [4,5]. Among these, PEID is a minimally invasive procedure that involves directly accessing the central spinal canal region via the ligamentum flavum window in the intervertebral foramen to achieve decompression. This technique avoids the iliac crest bone obstruction issues associated with the traditional transforaminal approach, enabling direct decompression of the central spinal canal [6,7,8]. It is considered a superior option for L5/S1 disc herniation surgery [9,10].

Traditional PEID procedures typically rely on identifying the “V-point” for localisation and working channel placement [11]. However, this approach often presents challenges when managing large central disc herniations, such as incomplete contralateral disc resection, prolonged operative time, and increased recurrence rates [12,13,14]. Additionally, the L5/S1 intervertebral space cannot frequently be positioned perpendicular to the ground. During surgery, traction is inevitably applied to the working channel, and the nerve root is compressed when the contralateral disc is managed, further increasing surgical risk [15,16,17].

To address these limitations, we refined the conventional PEID trajectory by utilising the inferolateral margin of the L5 pedicle projection as a modified cutaneous entry landmark. This trajectory adjustment aims to establish a disc-centred positioning logic and achieve a working angle parallel to the intervertebral disc, effectively preventing disorientation during surgery while expanding the endoscopic field of vision and operative space. It facilitates more thorough removal of deeper, softer nucleus pulposus material within the disc and enables access to the contralateral intervertebral space, while simultaneously reducing compression on the nerve roots. It is important to emphasise that this modification pertains exclusively to the entry point and approach vector; the internal decompression workflow (ligamentum flavum incision, laminotomy, discectomy, and nerve root decompression) follows established PEID principles. This consecutive retrospective cohort study evaluates the technical feasibility and short-term clinical and radiographic outcomes of a modified trajectory for L5/S1 disc herniation. Secondary objectives include: (1) comparing surgical efficiency and clinical outcomes between central and non-central herniation groups; and (2) determining whether central disc herniation independently predicts improved surgical efficiency after adjusting for confounders.

## 2. Methods

### 2.1. Study Design and Patient Population

This single-centre retrospective cohort study was approved by the Institutional Review Board of the authors’ institution (Approval Number: [LYZX-2025-207-01]). All participants signed informed consent forms.

### 2.2. Inclusion Criteria, Exclusion Criteria and Patients

Inclusion criteria: 1. Radicular leg pain with a positive straight leg raise test; 2. MRI (magnetic resonance imaging)-confirmed L5/S1 disc herniation; 3. failure of conservative treatment for more than 6 weeks.

Exclusion criteria: 1. History of L5/S1 spinal surgery; 2. significant lumbar instability or deformity; 3. spinal tumours, infections, tuberculosis, or trauma; 4. L5/S1 spinal canal stenosis requiring decompression treatment; 5. Michigan State University (MSU)-C type herniation (this type is frequently treated via a transforaminal approach at our centre).

On the basis of the inclusion and exclusion criteria, this study enrolled 70 patients with intervertebral disc herniation between June 2023 and July 2025. The cohort comprised 39 males and 31 females, with a mean age of 39 ± 1.5 years (range 16–65 years). On the basis of preoperative MRI findings, 32 patients were classified as having central herniation (MSU-A type) and 38 as having noncentral herniation (MSU-B type). The mean follow-up duration was 11.77 ± 0.8 months. All procedures were consistently performed by a senior attending physician with extensive experience in spinal endoscopy (having completed more than 2000 procedures).

### 2.3. Outcome Measurements

**Figure 1:**
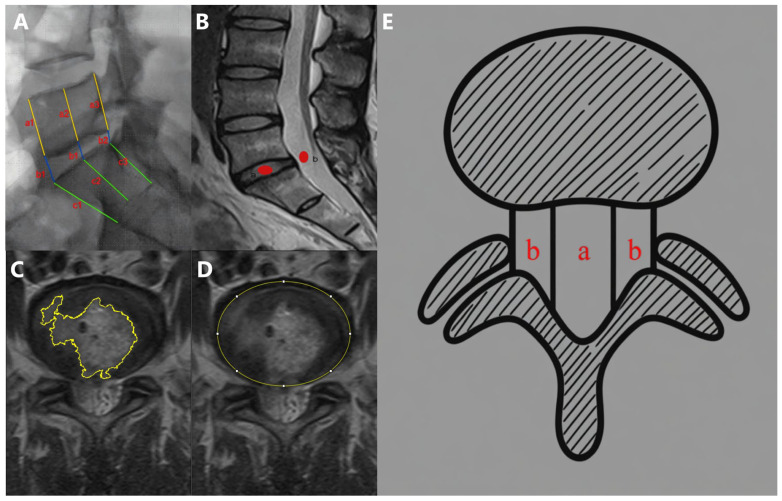
Schematic of imaging measurements. (**A**) Disc height index (DHI), DHI = [2(b1 + b2 + b3)]/[(a1 + a2 + a2) + (c1 + c2 + c3)] × 100%. (**B**) Ratio value of the greyscale (RVG). Midsagittal T2-weighted images were chosen, and RVG was the greyscale of discs, normalised against the greyscale of cerebrospinal fluid at the same level, a indicates the greyscale of the intervertebral disc, and b indicates the greyscale of the cerebrospinal fluid at the same level. RVG was calculated as the ratio of a to b. (**C**,**D**) Schematic diagram of measuring the disc resection area using ImageJ: Resected disc area = yellow-circled region in panel (**C**); resection percentage = yellow-circled region in panel (**C**); yellow-circled region in panel (**D**) × 100%. (**E**) Definition of central disc protrusion: The protrusion apex is located between the medial aspects of both pedicles, as shown in region a of the figure.

**Figure 2:**
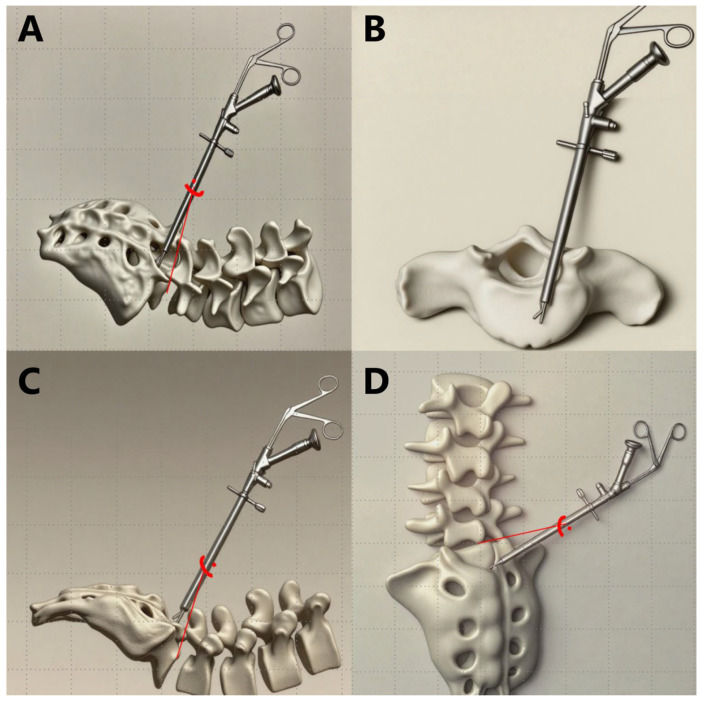
Schematic diagram of surgical approach. The red circle indicates the percutaneous entry point. The red line represents a line parallel to the L5/S1 intervertebral space, passing through the percutaneous entry point. (**A**) Lateral view; (**B**) axial view; (**C**) lateral distracted view; (**D**) posterior oblique view.

**Figure 3:**
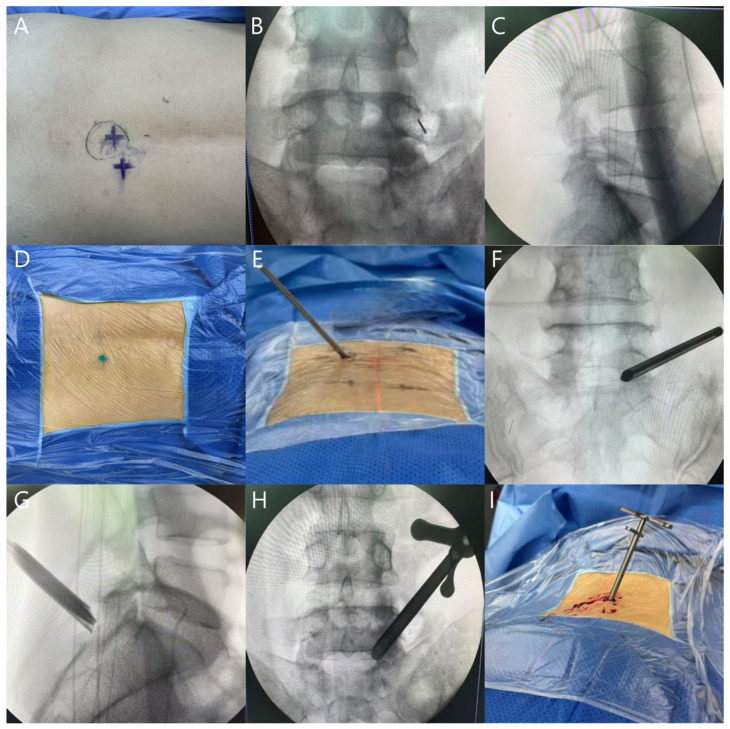
(**A**) Preoperative surface marking; (**B**) insertion localisation under AP (anteroposterior) fluoroscopy; (**C**) insertion under lateral fluoroscopy; (**D**) needle placement on oblique view; (**E**) dilator placement on oblique view; (**F**) dilator under AP fluoroscopy; (**G**) trocar insertion under lateral fluoroscopy; (**H**) trocar insertion under AP fluoroscopy; (**I**) trocar placement on oblique view.

**Figure 4:**
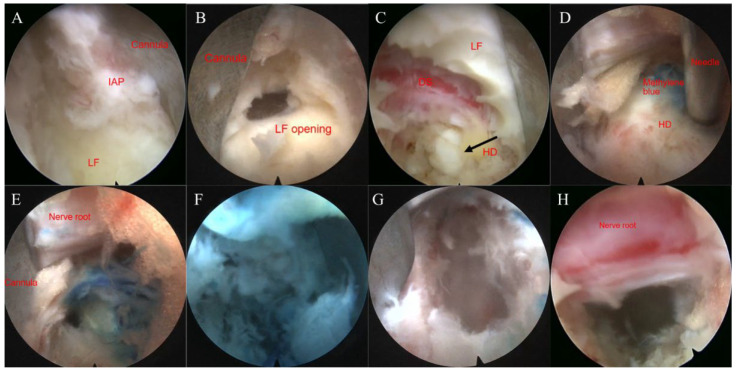
(**A**) Exposing the surface of the ligamentum flavum; (**B**) incising the ligamentum flavum; (**C**) exposing the herniated nucleus pulposus; (**D**) injecting methylene blue for discography; (**E**) accessing the intervertebral disc; (**F**) stained loose nucleus pulposus within the disc; (**G**) completion of discectomy; (**H**) visual confirmation of normal dural sac and nerve root pulsation.

This study adopted the Michigan State University (MSU) classification for disc herniation types [18]. The ratio value of the greyscale (RVG) was calculated via the method proposed by Li et al. [19]. Postoperative T2-weighted MR images were analysed via ImageJ software (version 1.54; National Institutes of Health, Bethesda, MD, USA) to measure the Postoperative Disc Resection Extent (DRE) of the L5/S1 disc (defined as the region showing signal intensity changes in the L5/S1 disc cross-section). The Disc Resection Proportion (DRP) was calculated as follows: Disc Resection Extent/Total Disc Extent (Figure 1); A schematic diagram of laminectomy for a herniated disc via the lateral pedicle approach is shown in Figure 2. The operative procedure is illustrated in Figure 3, and the endoscopic workflow is depicted in Figure 4.

### 2.4. Surgical Technique: Modified Trajectory Description

The following describes the modified entry trajectory. The internal decompression steps (laminotomy, ligamentum flavum management, discectomy, and nerve root decompression) follow standard PEID principles [20].

#### 2.4.1. Step 1: Positioning and Anaesthesia

All surgical patients were positioned prone on the radiosurgical table with a slight head-high, feet-low inclination, and the abdomen was suspended. General anaesthesia is administered; for patients intolerant to general anaesthesia, regional anaesthesia is used.

#### 2.4.2. Step 2: Localisation and Puncture (Modified Trajectory)

Adjustment of the C-arm fluoroscopy angle enables alignment of L5 and S1 endplates, respectively, without bilateral signs. The L5 pedicle was located, and the puncture entry point at the lateral inferior border of the L5 pedicle was marked. A 22 G, 25 mm needle was inserted vertically from the entry point to the surface of the articular process. Under anteroposterior and lateral X-ray, the segment that intersects the L5/S1 intervertebral space extension line at the entry point was confirmed.

This entry point differs from conventional PEID, which typically uses the “V-point” (junction of the superior articular process and lamina) as the primary landmark. Our modified entry point is located more laterally and inferiorly relative to the conventional V-point, creating a more anterolateral working angle toward the disc space (Figure 2).

#### 2.4.3. Step 3: Exposure and Canal Entry

A 0.7 cm longitudinal incision was made at the puncture site. Following dilator insertion and working channel placement (with the trocar tip positioned at the superior border of the S1 lamina at the ligamentum flavum junction), the endoscope was introduced. The “white-yellow” junction between the ligamentum flavum and lamina was identified and dissected laterally to locate the medial border of the S1 superior articular process.

The method of spinal canal entry was selected based on the preoperative lamina gap size: ligamentum flavum incision for wide gaps, or lateral recess incision for narrow gaps. Both techniques follow standard PEID protocols. The lateral recess approach involves burr-assisted laminotomy at the medial margin of the superior articular process, ligamentum flavum incision, and partial removal to expose the dura mater and nerve root.

#### 2.4.4. Step 4: Discectomy and Nerve Root Decompression

Following standard laminotomy, the relationship between the nerve root and herniation was identified. The nerve root was carefully retracted to expose the protruding nucleus pulposus. For cases without clearly visible rupture, methylene blue discography was performed (1 mL methylene blue + 9 mL 0.9% saline) to identify loose fragments. The operative endpoint was confirmed by spontaneous pulsation of the dural sac and nerve roots, absence of active bleeding after irrigation cessation, and unobstructed nerve root mobility.

### 2.5. Data Collection and Statistical Analysis

Data including gender, age, height, weight, disease duration, operative time, intraoperative blood loss, haemoglobin drop, complications, and length of hospital stay were collected through the medical record system. Low back and leg pain were assessed using the visual analogue scale (VAS) and the Japanese Orthopaedic Association (JOA) score preoperatively, on postoperative day 1, at 3 months postoperatively, and at the final follow-up. The modified MacNab criteria were applied to evaluate clinical efficacy at the final follow-up.

Statistical analysis was performed using SPSS 26.0 (IBM Corp., Armonk, NY, USA). Quantitative data following normal distribution are presented as mean ± SD; otherwise, as median (IQR). Between-group comparisons used independent samples *t*-test or Mann–Whitney U test as appropriate. Categorical data were compared using Pearson’s chi-square or Fisher’s exact test. Multivariable linear regression was performed to adjust for potential confounders (age, BMI, disease duration, herniation type) when examining associations with operative time, blood loss, and DRE. *p* < 0.05 was considered statistically significant.

## 3. Results

### 3.1. Demographics and Surgical Parameters

**Figure 5:**
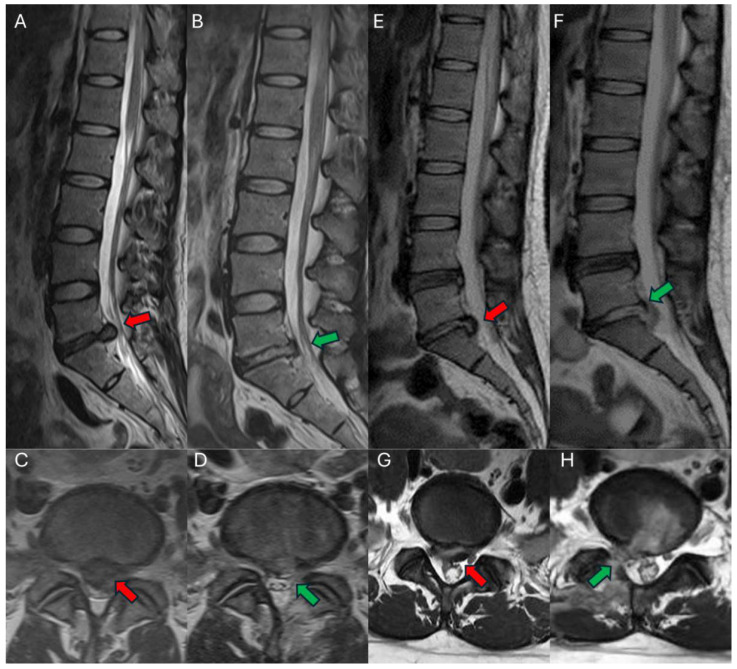
(**A**–**D**) Case 1: 35-year-old male. (**E**–**H**) Case 2: 39-year-old female. Red arrows indicate preoperative MR images; green arrows indicate postoperative MR images following PEID.

All 70 patients underwent successful surgery. The mean operative time was 102.5 (75, 131.2) minutes, with a median blood loss of 10 (5, 10) mL. The mean postoperative hospital stay was 3 (2, 4) days. Compared with the non-central herniation group, the central herniation group demonstrated a significantly shorter operative time (*p* = 0.027), reduced blood loss (*p* = 0.047), and a smaller decrease in haemoglobin (*p* = 0.033). Postoperative MRI analysis revealed an average DRE of 572.85 ± 17.18 mm^2^ and DRP of 36.6% ± 0.007%. No significant difference in the DRE was observed between the central and noncentral types (*p* = 0.378) (Table 1). Postoperative MR confirmed the complete removal of the herniated disc, with adequate decompression of the compressed dural sac and displaced nerve roots (Figure 5).

### 3.2. Clinical Outcomes

**Figure 6:**
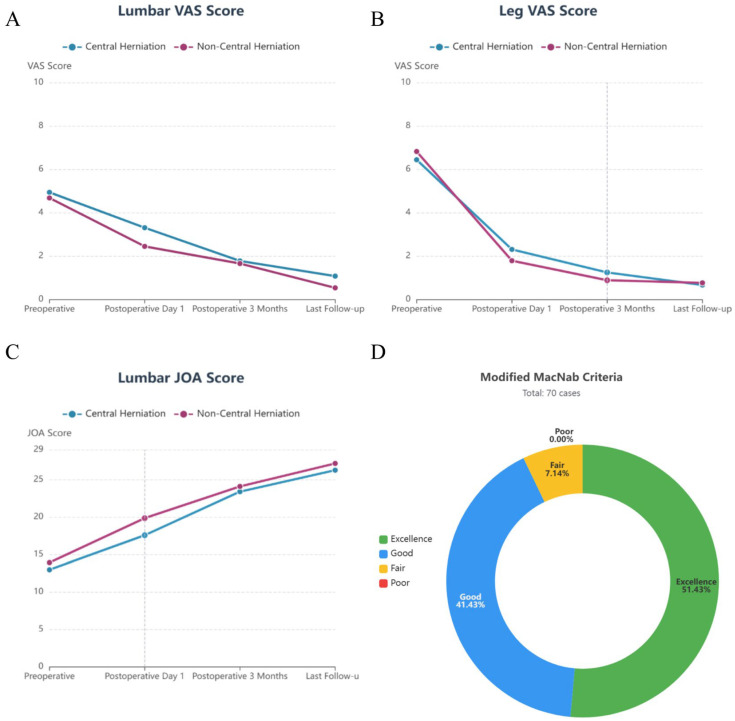
Clinical outcomes at different follow-up stages from preoperation to last follow-up (**A**) visual analogue scale (VAS) lumber pain scores; (**B**) visual analogue scale (VAS) Leg pain scores; (**C**) Japanese Orthopaedic Association Score of lumber; (**D**) modified MacNab criteria at the last follow-up.

Both patient groups demonstrated significant improvements in VAS low back pain scores, VAS leg pain scores, and JOA scores at all postoperative time points (*p* < 0.001). Except for superior JOA scores in the non-central herniation group on postoperative day 1 (*p* = 0.047), no statistically significant intergroup differences were observed at the other time points (Table 2). At the final follow-up (mean 11.77 ± 0.8 months), the modified MacNab criteria were excellent in 36 patients (51.4%), good in 29 patients (41.4%), fair in 5 patients (7.1%), and not poor. The overall satisfaction rate reached 92.9% (Figure 6).

### 3.3. Linear Regression Analysis

Multivariable linear regression analysis identified central disc herniation as an independent predictor of improved surgical efficiency. After adjusting for sex, age, BMI, disease duration, and DRE, the central herniation group was significantly associated with shorter operative time (*p* = 0.032), reduced intraoperative blood loss (*p* = 0.033), and decreased haemoglobin drop (*p* = 0.004). Longer disease duration independently predicted increased operative time (*p* = 0.036), whereas male sex (*p* = 0.006) and larger DRE (*p* = 0.023) were independently associated with greater haemoglobin drop (Table 3).

### 3.4. Complications and Reoperations

Four complications occurred (5.7%): transient sensory dullness in 2 patients (2.9%), dural tear in 1 patient (1.4%), and recurrent disc herniation requiring revision surgery in 1 patient (1.4%). The dural tear was repaired intraoperatively with fibrin glue; no cerebrospinal fluid leakage occurred after 72 h of bed rest. The recurrent herniation occurred in a non-central herniation group patient at 1 month postoperatively and was managed with minimally invasive fusion surgery. The 5.7% complication rate is within the range reported for conventional PEID in the literature (3.6–12.8%) [21,22]. No major vascular or neural injuries occurred.

## 4. Discussion

This retrospective pilot study evaluated a modified PEID trajectory utilising the inferolateral margin of the L5 pedicle projection as an alternative entry landmark for L5/S1 disc herniation. Our findings demonstrate that this modified trajectory is technically feasible and was associated with favourable short-term clinical outcomes, including a 92.9% satisfaction rate and significant improvements in VAS and JOA scores.

PEID has become an established treatment for L5/S1 lumbar disc herniation (LDH). However, traditional interlaminar approaches often rely on the “V-point” as the primary anatomical landmark [23], which can be suboptimal for central disc herniations because the L5/S1 interspace is frequently angled relative to the horizontal plane. In such scenarios, accessing the central canal and contralateral disc space often requires exaggerated angulation of the endoscope and greater manipulation of the dural sac and nerve root, potentially increasing operative time, blood loss, and the risk of incomplete decompression [24,25,26]. The present study describes a disc-centred trajectory modification for PEID at L5/S1, using the lateral inferior margin of the L5 pedicle projection as the cutaneous entry landmark. The intent of this modification is to establish an initial working channel that is more perpendicular to the disc space and closer to the midline, thereby facilitating direct visualisation of the central canal and minimising the need for aggressive nerve root retraction when addressing central herniations.

It is important to clarify the nature and scope of this technical modification. The current approach does not represent a fundamentally new decompressive strategy; rather, it is a refinement of the access geometry. The internal bony and soft-tissue workflow—laminotomy at the lateral recess, partial medial facetectomy when necessary, ligamentum flavum incision, and discectomy—remains consistent with conventional PEID principles. What differs is the initial puncture trajectory and the working angle: the entry point is deliberately set at the intersection of the interspinous line and the lateral inferior border of the L5 pedicle projection, and the guide rod is oriented with its tail parallel to the L5/S1 interspace. This creates a more cranial-to-cauda, near-vertical working axis that aligns more closely with the physiological orientation of the intervertebral disc. During initial trocar insertion, the tip is positioned at the superior border of the S1 lamina and then advanced cranially toward the ligamentum flavum junction, establishing a trajectory parallel to the L5/S1 interspace. Consequently, the endoscope approaches the central canal from a slightly anterolateral angle, providing a direct view of the medial facet joint and the axillary region of the nerve root. For central herniations, this trajectory may reduce the degree of endoscope angulation required to reach the contralateral disc space, potentially lessening traction on the traversing nerve root. For paracentral herniations, we did not employ a more medial skin incision, as this approach increases paraspinal muscle dissection and soft tissue resistance, resulting in greater endoscope-medial facet contact, restricted instrument manoeuvrability, and reduced effective working space. Instead, we performed a limited partial medial facetectomy (resecting <50% of the medial superior articular process of S1) using a 3 mm diamond burr to expose the lateral dural margin, while preserving >50% of the lateral facet to maintain segmental stability. Nevertheless, when the congenital lateral recess is narrow, partial resection of the medial superior articular process of S1 or the cranial margin of the S1 lamina is still required to establish adequate working space; therefore, claims of reduced bony resection should be interpreted cautiously and require prospective volumetric comparison against standard PEID in future studies. Furthermore, the working channel established by this approach facilitates direct endoscopic management of the intervertebral space and implantation of fusion devices, laying the groundwork for expanded applications of endoscopic fusion surgery.

While previous studies have described endoscopic approaches for lumbar spinal stenosis, our modified interlaminar trajectory may offer certain technical considerations in selected cases. The L5/S1 foramen is inherently narrow and frequently complicated by iliac crest obstruction, making transforaminal navigation technically demanding and increasing the risk of exiting nerve root compression during instrumentation. Lewandrowski et al. reported that central stenosis or complex foraminal lesions should be considered as alternative indications for transforaminal decompression, highlighting the anatomical limitations of this approach in accessing central pathology [27]. In contrast, our interlaminar entry completely avoids the neuroforamen, eliminating the need for foraminoplasty or angled navigation that jeopardises the exiting nerve root, while providing direct perpendicular access to the central canal. Sairyo et al. developed transforaminal full-endoscopic lumbar undercutting laminectomy (TE-LUL) for central canal stenosis under local anaesthesia, demonstrating the feasibility of transforaminal central decompression through total removal of the superior articular process and partial resection of the inferior articular process; however, from ref. [28], this technique requires extensive facet joint sacrifice and carries potential instability risks. Our uniportal interlaminar technique achieves comparable central canal visualisation through a single minimally invasive portal while preserving posterior spinal elements and creating a more perpendicular working angle specifically optimised for central lesions. Ju et al. established that the interlaminar approach is preferred for bilateral central stenosis and lateral recess decompression, whereas the transforaminal approach is contraindicated for multiple spinal stenoses, bilateral symptoms, and high iliac crest cases due to anatomical limitations [29]. However, our study further refines this concept by introducing a modified pedicle-projection landmark that provides a reproducible, disc-centred entry point, potentially reducing the learning curve and improving operative efficiency specifically for central lesions.

In this retrospective cohort, the central herniation group exhibited shorter operative time, lower intraoperative blood loss, and smaller postoperative haemoglobin drops compared with the non-central herniation group. While these differences were statistically significant, their clinical magnitude was modest, and the absence of a control group treated with standard PEID precludes definitive attribution of these benefits to the modified trajectory alone. Moreover, the two groups achieved comparable long-term VAS and JOA improvements, and the modified MacNab satisfaction rates were similar (89.47% vs. 96.87%, *p* = 0.411).

Multivariable regression (Table 3) demonstrated that the central herniation group maintained independent advantages in operative efficiency after adjusting for confounders. This indicates that the intergroup differences observed in Table 1 cannot be explained by confounding factors such as age, BMI, or disease duration but are more likely related to the improved approach’s anatomical compatibility with central protrusions—this trajectory positions the working channel closer to the centre of the intervertebral disc, reducing additional manoeuvres and instrument adjustments required to access the contralateral region. Notably, resected disc area correlated only with haemoglobin drop (*p* = 0.023), not with operative time or blood loss, while male sex predicted greater haemoglobin drop (*p* = 0.006), likely reflecting higher baseline values. Nevertheless, the low R^2^ values (0.104–0.254) indicate substantial unexplained variance, and postoperative haemoglobin changes are confounded by intraoperative fluid dilution; these findings should be interpreted cautiously and validated in prospective controlled studies.

### Limitations

This study has several limitations. First, it is a retrospective, single-centre study with a relatively small sample size (*n* = 70) and no concurrent control group treated with standard PEID. Without such a comparison, we cannot determine whether the observed reductions in operative time and blood loss are attributable to the modified projection, surgeon experience, or selection bias. Second, postoperative haemoglobin decline was likely confounded by intraoperative fluid haemodilution rather than true blood loss, given its disproportionality to measured intraoperative bleeding, though the significant intergroup difference may still reflect differential surgical trauma. Third, the mean follow-up of approximately 12 months is insufficient to assess long-term outcomes such as recurrence rates, adjacent-segment degeneration, or lumbar stability. Third, we did not perform quantitative volumetric analysis of bone removal (e.g., facet joint resection volume), which limits objective assessment of whether this approach truly reduces bony resection. Finally, no biomechanical or kinematic data (e.g., flexion–extension radiographs) were collected postoperatively. Prospective, multicenter, randomised controlled studies with longer follow-up and volumetric assessment of bony resection are warranted to validate its specific advantages over standard PEID.

## 5. Conclusions

Percutaneous endoscopic interlaminar discectomy via the L5 pedicle projection lateral inferior margin approach is a technically feasible modification that optimises the initial working trajectory toward the central disc space. For central L5/S1 disc herniation, this disc-centred alignment may facilitate operative efficiency and reduce intraoperative blood loss, though clinical outcomes remain comparable to those of non-central herniations. The modified trajectory follows a more anterolateral path through the paraspinal musculature, reducing soft tissue resistance and enabling smoother instrument manipulation. Furthermore, the parallel-to-disc working angle naturally aligns the working channel with the intervertebral space, providing favourable access for deeper nucleus pulposus exploration and positioning this technique as a potential precursor to fully endoscopic interbody fusion procedures.

## Figures and Tables

**Table 1:** Baseline characteristics of the two groups.

Characteristics	Non-Central (*n* = 38)	Central (*n* = 32)	*p* Value
Gender, *n* (%)			0.377
Male	23 (60.53%)	16 (50.00%)	
Female	15 (39.47%)	16 (50.00%)	
Symptomatic limb *n* (%)			0.036 *
Left	18 (47.37%)	14 (43.75%)	
Right	19 (50.00%)	11 (34.38%)	
Bilateral	1 (2.63%)	7 (21.88%)	
Age (years)	37.84 ± 11.45	40.75 ± 13.70	0.337
BMI (kg/m^2^)	24.42 ± 4.10	23.53 ± 3.26	0.328
Disease duration (months)	7.5 (3.0, 24.25)	8.5 (4.0, 60.5)	0.239
Follow-up time (months)	9.5 (5.75, 18.25)	11.0 (6.0, 17.5)	0.958
Postoperative length of stay (days)	3.0 (2.0, 4.0)	3.0 (2.0, 4.0)	0.856
Intraoperative blood loss (mL)	10.0 (10.0, 20.0)	10 (5.0, 10.0)	0.047 *
Operative time (minutes)	115.0 (80.0, 141.25)	85.0 (62.5, 118.75)	0.027 *
Postoperative haemoglobin drop (g/L)	9.00 ± 8.34	4.53 ± 8.83	**0.033** *
RVG	0.22 ± 0.07	0.23 ± 0.09	0.418
DRE (mm^2^)	558.86 ± 126.26	589.48 ± 162.56	0.378
DRP (%)	0.36 ± 0.06	0.37 ± 0.07	0.581
Pfirrmann_Grading (1/2/3/4/5)	0/11/25/2/0	0/11/18/3/0	0.660
Weishauptgrade (0/1/2/3)	1/31/5/1	5/20/7/0	0.116

Data are presented as mean ± standard deviation, median (interquartile range), or n (%). BMI = Body Mass Index; postoperative haemoglobin drop = postoperative day 1 HB—preoperative HB; RVG = ratio value of the greyscale; DHI = disc height index; DRE = disc resection extent; DRP = disc resection proportion. * *p* < 0.05 represents a statistical difference.

**Table 2:** Comparison of clinical efficacy of functional scores data between the two groups.

	Non-Central (*n* = 38)	Central (*n* = 32)	*p* Value
Preop VAS—low back pain	5.0 (1.0, 7.25)	6.0 (2.0, 7.0)	0.877
Preop VAS—leg pain	7.0 (6.0, 8.0)	7.0 (5.0, 8.0)	0.815
Preop JOA score	13.92 ± 4.75	12.97 ± 4.11	0.378
Postop 1 day VAS—low back pain	2.0 (1.0, 4.0)	3.0 (2.0, 4.0)	0.071
Postop 1 day VAS—leg pain	1.5 (0, 3.0)	2.0 (1.0, 3.0)	0.268
Postop 1 day JOA score	19.84 ± 4.90	17.56 ± 4.44	0.047 *
Postop 3 months VAS—low back pain	1.5 (0, 3.0)	2.0 (0.25, 2.75)	0.575
Postop 3 months VAS—leg pain	0.0 (0, 2.0)	1.0 (0, 1.75)	0.606
Postop 3 months JOA score	25.0 (21.0, 27.0)	24.0 (21.0, 25.75)	0.384
Last follow-up VAS—low back pain	0.5 (0, 2.0)	0 (0, 2.0)	0.877
Last follow-up VAS—low leg pain	0 (0, 1.0)	0 (0, 1.0)	0.807
Last follow-up JOA score	26.0 (25.0, 28.0)	27.0 (24.25, 28.75)	0.900
MacNab evaluation *n* (%)			0.411
Excellence	20 (52.63)	16 (50.00)	
Good	14 (36.84)	15 (46.88)	
Fair	4 (10.53)	1 (3.13)	
Poor	0 (0)	0 (0)	
Excellence/good rate	89.47%	96.87%	
Complications			0.704
Recurrent disc herniation	1	0	
Dural sac tear	1	0	
Paraesthesia	1	1	

VAS = visual analogue scale; JOA = Japanese Orthopaedic Association Score. * *p* < 0.05 represents a statistical difference.

**Table 3:** Multivariable linear regression analysis of surgical efficiency outcomes.

	Operative Time (Modle.1)				IBL (Modle.2)				Hbdrop (Modle.3)			
	B	*p*	LCL	UCL	B	*p*	LCL	UCL	B	*p*	LCL	UCL
Costent	74.582	0.164	−31.227	180.392	12.259	0.042 *	0.470	24.047	8.342	0.354	−9.529	26.214
Sex	−0.604	0.965	−28.006	26.799	0.135	0.930	−2.918	3.188	6.575	0.006 *	1.946	11.203
Age	−0.123	0.819	−1.193	0.947	0.047	0.432	−0.072	0.166	−0.102	0.265	−0.283	0.079
BMI	0.982	0.565	−2.415	4.380	0.042	0.824	−0.336	0.421	−1.280	0.205	−0.941	0.206
Disease Duration	0.290	0.036 *	0.019	0.561	0.003	0.839	−0.027	0.033	0.007	0.776	−0.039	0.052
Group	−25.352	0.032 *	−48.417	−2.270	−2.809	0.033 *	−5.378	−0.239	−5.812	0.004 *	−9.708	−1.916
DRE	0.035	0.473	−0.062	0.133	−0.006	0.251	−0.017	0.005	0.019	0.023 *	0.003	0.036

IBL = intraoperative blood loss; Hbdrop = postoperative haemoglobin drop; B = unstandardised regression coefficient; LCL = lower confidence limit; UCL = upper confidence limit. BMI = Body Mass Index; DRE = disc resection extent. * *p* < 0.05 represents a statistical difference; Modle.1: R^2^ = 0.137; Modle.2: R^2^ = 0.104; Modle.3: R^2^ = 0.254.

## Data Availability

The data sets used and analysed during the current study are available from the corresponding author on reasonable request.

## References

[1] Amin R.M., Andrade N.S., Neuman B.J. (2017). Lumbar disc herniation. Curr. Rev. Musculoskelet. Med..

[2] Ravindra V.M., Senglaub S.S., Rattani A., Dewan M.C., Härtl R., Bisson E., Park K.B., Shrime M.G. (2018). Degenerative lumbar spine disease: Estimating global incidence and worldwide volume. Glob. Spine J..

[3] Pojskic M., Bisson E., Oertel J., Takami T., Zygourakis C., Costa F. (2024). Lumbar disc herniation: Epidemiology, clinical and radiologic diagnosis WFNS spine committee recommendations. World Neurosurg. X.

[4] Song K., Liang J., Zhang M., Cai S., Wang Y., Wu W. (2025). Comparison of different treatments for lumbar disc herniation: A network meta-analysis and systematic review. BMC Surg..

[5] Patel T., Farhan M., Nashaat Hamza D., Daneshpazhouh M., Nahar O.A., Serafy A.E., Mohamed Hasan A.G., Bnaian L., Ali M.H., Sobhi A. (2025). Comparative effectiveness of minimally invasive endoscopic discectomy versus conventional surgical techniques for lumbar disc herniation: A systematic review and meta-analysis. Ann. Med. Surg..

[6] Liu S., Zhang X., Xiong Y., He H. (2025). Minimally invasive surgery for lumbar disc herniation: A meta-analysis of efficacy and safety. Int. J. Surg..

[7] Wu T.-L., Yuan J.-H., Jia J.-Y., He D.-W., Miao X.-X., Deng J.-J., Cheng X.-G. (2021). Percutaneous endoscopic interlaminar discectomy via laminoplasty technique for L5 -S1 lumbar disc herniation with a narrow interlaminar window. Orthop. Surg..

[8] Tang Y., Li H., Qin W., Liu Z., Liu H., Zhang J., Mao H., Zhang K., Chen K. (2023). Comparison of percutaneous endoscopic interlaminar discectomy and conventional open lumbar discectomy for L4/5 and L5/S1 double-segmental lumbar disk herniation. J. Orthop. Surg. Res..

[9] Zhang H., Gao J. (2025). Clinical comparison of percutaneous endoscopic lumbar discectomy and posterior lumbar interbody fusion for L4/5 and L5/S1 dual-level disc herniation. Sci. Rep..

[10] Song H., Hu W., Liu Z., Hao Y., Zhang X. (2017). Percutaneous endoscopic interlaminar discectomy of L5-S1 disc herniation: A comparison between intermittent endoscopy technique and full endoscopy technique. J. Orthop. Surg. Res..

[11] Lee J.-S., Kim H.-S., Jang J.-S., Jang I.-T. (2016). Structural preservation percutaneous endoscopic lumbar interlaminar discectomy for L5-S1 herniated nucleus pulposus. BioMed Res. Int..

[12] Choi K.-C., Lee J.-H., Kim J.-S., Sabal L.A., Lee S., Kim H., Lee S.-H. (2015). Unsuccessful percutaneous endoscopic lumbar discectomy: A single-center experience of 10 228 cases. Neurosurgery.

[13] Yin S., Du H., Yang W., Duan C., Feng C., Tao H. (2018). Prevalence of recurrent herniation following percutaneous endoscopic lumbar discectomy: A meta-analysis. Pain Physician.

[14] Xiao L., Zhou J., Zhong Q., Zhang X., Cao X. (2025). Clinical comparison of percutaneous endoscopic interlaminar vs. unilateral biportal endoscopic discectomy for lumbar disc herniation: A retrospective study. Sci. Rep..

[15] Qu X., Zhang L., Xie Z., Zhang J., Huang Y., Li N., Luo X. (2024). Efficacy of endoscopic interlaminar decompression in lumbar spinal stenosis: A retrospective study. Sci. Rep..

[16] Kim J.Y., Kim H.S., Jeon J.B., Lee J.H., Park J.H., Jang I.-T. (2021). The novel technique of uniportal endoscopic interlaminar contralateral approach for coexisting L5-S1 lateral recess, foraminal, and extraforaminal stenosis and its clinical outcomes. J. Clin. Med..

[17] Gollogly S., Yue J., Van Isseldyk F., Kim J.-S., Farshad M. (2024). Endoscopic contralateral transaxillary discectomy for recurrent disc herniation. Neurospine.

[18] Mysliwiec L.W., Cholewicki J., Winkelpleck M.D., Eis G.P. (2010). MSU classification for herniated lumbar discs on MRI: Toward developing objective criteria for surgical selection. Eur. Spine J..

[19] Li T., Yang G., Zhong W., Liu J., Ding Z., Ding Y. (2024). Percutaneous endoscopic transforaminal vs. interlaminar discectomy for L5-S1 lumbar disc herniation: A retrospective propensity score matching study. J. Orthop. Surg. Res..

[20] Nilmart P., Vongsirinavarat M. (2026). Co-occurrence of suspected scoliosis and sagittal spinal deviations among early adolescents: A school-based cross-sectional study of prevalence and associated factors. Front. Pediatr..

[21] Zhao K., Li L.-D., Li T.-T., Xiong Y. (2022). Percutaneous endoscopic lumbar discectomy for the treatment of recurrent lumbar disc herniation: A meta-analysis. BioMed Res. Int..

[22] Chen X., Chamoli U., Vargas Castillo J., Ramakrishna V.A.S., Diwan A.D. (2020). Complication rates of different discectomy techniques for symptomatic lumbar disc herniation: A systematic review and meta-analysis. Eur. Spine J. Off. Publ. Eur. Spine Soc. Eur. Spinal Deform. Soc. Eur. Sect. Cerv. Spine Res. Soc..

[23] Kim H.-S., Wu P.H., Jang I.-T. (2020). Lumbar endoscopic unilateral laminotomy for bilateral decompression outside-In approach: A proctorship guideline with 12 steps of effectiveness and safety. Neurospine.

[24] Gao A., Yang H., Zhu L., Hu Z., Lu B., Jin Q., Wang Y., Gu X. (2020). Comparison of interlaminar and transforaminal approaches for treatment of L5/S1 disc herniation by percutaneous endoscopic discectomy. Orthop. Surg..

[25] Tezuka F., Sakai T., Abe M., Yamashita K., Takata Y., Higashino K., Chikawa T., Nagamachi A., Sairyo K. (2017). Anatomical considerations of the iliac crest on percutaneous endoscopic discectomy using a transforaminal approach. Spine J. Off. J. N. Am. Spine Soc..

[26] Chen K.-T., Wei S.-T., Tseng C., Ou S.-W., Sun L.-W., Chen C.-M. (2020). Transforaminal endoscopic lumbar discectomy for L5–S1 disc herniation with high iliac crest: Technical note and preliminary series. Neurospine.

[27] Lewandrowski K.-U., Dowling Á., Lee J.H., Burkhardt B.W., Alves Ó.L., Parajón A., Ramirez J.F., Montemurro N., Vaccaro A.R., Lorio M.P. (2026). Transforaminal endoscopic lumbar decompression: Defining its scope and limitations. Int. J. Spine Surg..

[28] Sairyo K., Yamashita K., Manabe H., Ishihama Y., Sugiura K., Tezuka F., Takata Y., Sakai T., Omichi Y., Takamatsu N. (2019). A novel surgical concept of transforaminal full-endoscopic lumbar undercutting laminectomy (TE-LUL) for central canal stenosis of the lumbar spine with local anesthesia: A case report and literature review. J. Med. Investig..

[29] Ju C.-I., Kim P., Ha S.-W., Kim S.-W., Lee S.-M. (2022). Contraindications and complications of full endoscopic lumbar decompression for lumbar spinal stenosis: A systematic review. World Neurosurg..

